# The epidemiology and management of stroke in French Guiana

**DOI:** 10.1186/s12883-020-01650-2

**Published:** 2020-03-24

**Authors:** Dévi Rita Rochemont, Emmanuelle Mimeau, Caroline Misslin-Tritsch, Martine Papaix-Puech, Emmanuel Delmas, Yannick Bejot, Bertrand DeToffol, Isabelle Fournel, Mathieu Nacher

**Affiliations:** 1 CIC INSERM 1424, Clinical Investigation Center, Cayenne general hospital, Cayenne, French Guiana; 2 Emergency, Cayenne hospital, Cayenne, French Guiana; 3 Department of Medicine, Centre Hospitalier de l’Ouest Guyanais, Saint Laurent du Maroni, French Guiana; 4 Emergency, Kourou Hospital, Kourou, French Guiana; 5 CIC INSERM 1432, Clinical Epidemiology Unit, University hospital of Dijon, Dijon, France; 6 CIC INSERM 1432, Clinical Investigation Center, University hospital of Dijon and Bourgogne university, UFR des sciences de santé, Dijon, France; 7 Dijon stroke Registry, EA7460, Department of Neurology, University Hospital and Medical School of Dijon, University of Burgundy, Dijon, France; 8 Department of Neurology, Cayenne general hospital, Cayenne, French Guiana; 9 University of French Guiana and CIC INSERM 1424, Clinical Investigation Center, Cayenne general hospital, Cayenne, French Guiana

**Keywords:** Stroke, French Guiana, Epidemiology, France, Emergency care

## Abstract

**Background:**

French Guiana has the highest incidence of ischemic and hemorrhagic stroke of all French territories. However, there is no further information on the epidemiology and management of stroke in French Guiana. Our goal was to describe the characteristics of patients in French Guiana in order to generate hypotheses regarding the determinants explaining the magnitude of this public health problem.

**Methods:**

We used the data of the French multicentre INDIA prospective cohort study which included consecutive patients aged > 18 years with a first-ever stroke from June 2011 to October 2014. For the present study, only patients with ischemic or hemorrhagic stroke admitted in one of the 3 participating hospitals were analyzed.

**Results:**

Among the 298 patients (mean age 62.2 ± 14.5 years, 63.7% man) included in French Guiana, 52% were born abroad. Most strokes were ischemic strokes (79%), 14% of which were thrombolyzed. Hypertension (70.2%), history of smoking (22%) and diabetes (25%) were the most common risk factors and 28.4% of patients had known but untreated hypertension. Overall 89 (38%) patients with ischemic stroke were admitted less than 4.5 h after the first symptoms. In-hospital mortality was greater for intracerebral hemorrhage (18.7%) than for ischemic stroke (4.2%). Overall, 84.5% had health insurance coverage and among these, 41.9% had CMU, the universal health insurance for the poor.

**Conclusions:**

The present study is the first epidemiologic description of stroke in French Guiana. The comparisons of these results show that stroke patients in French Guiana are epidemiologically atypical because they are younger, and more likely to be males than patients in mainland France. Stroke risk factors and delay between stroke and hospital admission were comparable with what is observed in France, suggesting that efforts should focus on primary care and social inequalities of health to alleviate the main determinants of stroke in French Guiana.

## Background

Strokes represent a major cause of death, disability and dementia worldwide [1–3]. In Europe, strokes cause over 1 million deaths, and worldwide, strokes cause 6.5 million deaths [4, 5]. The major stroke risk factors – both for ischemic and hemorrhagic stroke- are well known [6, 7], and it has been estimated that over 90% of the burden of disease is attributable to modifiable factors [7]. Among these, interventions targeting modifiable factors have shown their significant impact in reducing the incidence and mortality of strokes [8].

French Guiana is a French territory in South America situated between Brazil and Suriname. Although French Guiana benefits from a universal health care system just like mainland France, the administrative goal of equity struggles with the field reality of French Guiana: Health professional density is a third of the average in mainland France and access to care is thus generally more difficult with geographical heterogeneities. Despite the young age of the population (median 23 years), there has been an epidemiologic transition from tropical infectious diseases towards chronic diseases. Hence, the prevalence of obesity and diabetes is increasing and among the highest in France [9, 10]. Similarly, high blood pressure is also more prevalent than in mainland France [11, 12]. French Guiana is also marked by socio-economic difficulties, which may lead to social inequalities regarding health and access to care [13, 14].

Although smoking is significantly less frequent than in mainland France [15], the other risk factors for stroke are frequent in French Guiana and it has been shown that in French Guiana the estimated standardized incidence of stroke, both ischemic (189.5 per 100,000) and hemorrhagic (65.7 per 100,000), is the highest in France [1]. Cerebrovascular diseases are among the top 5 causes of premature death (death before 65 years) in French Guiana [16].

Despite these figures, there have been no specific studies on strokes in French Guiana. The epidemiological data on strokes in France originates from urban registries in mainland France only. Given the high incidence of strokes in French Guiana, and the lack of epidemiological description, it seemed important to describe the characteristics of patients in French Guiana in order to generate hypotheses regarding the determinants explaining the magnitude of this public health problem. The objective of the present study was to describe the epidemiology and characteristics of stroke in French Guiana, and its outcomes, using a cross-section of the data of the INDIA (INégalités sociales et pronostic des accidents vasculaires cérébraux à DIjon et en Antilles-Guyane) cohort study.

## Methods

### Study design

The design was a cross-sectional analysis of prospective data collection from patients presenting a first episode of stroke in French Guiana described in detail in [17]. Consecutive patients aged > 18 years admitted for an acute stroke, confirmed by neuroimaging, and who were able to be interviewed either personally or via a next of kin were eligible. Exclusion criteria were a history of symptomatic stroke, presence of other short-term life-threatening diseases and inability to contact patients (or support persons) by telephone during follow-up. Between June 2011 and October 2014, 301 patients were recruited in emergency or medicine departments of three hospitals in French Guiana (Cayenne, Saint-Laurent du Maroni, and Kourou). Among them, patients with Subarachnoid Hemorrhage (SAH) (*n* = 3) were excluded. For the present study, only baseline and hospital stay data were considered. According to French law relative to observational studies, all patients or their next of kin received written information about the study but signed consent was not required. The study protocol was approved by the Burgundy Ethics Committee (CPP Est 1, 16 May 2010) and the National Commission for Data Processing and Civil Liberties (15 April 2011).

### Collected data

Demographic data, preexisting conditions, pre-stroke modified Rankin scale (mRS), admission NIHSS score, which categorizes stroke severity upon patient arrival, and mechanism of stroke were collected at the time of inclusion. The mRs and NIHSS scores were also collected after discharge. Anamnestic information included medications, history of ischemic vascular diseases, known and suspected cardiovascular risk factors. Patients, or their next of kin, were interviewed on their socio economic status, on the geographic origin of their parents or ancestors, and on their marital status employment status, education, housing conditions, wealth indicators, type of health insurance and deprivation defined by an EPICES score > 30.17. They were also interviewed on healthcare access including the travel time to the nearest primary care physician (PCP), the travel time to the nearest hospital, health insurance, the number of consultations with a PCP, a dentist or a specialist physician within the previous year, or a previous hospitalization > = 24 h in the 12 preceding months. Stroke therapies (including thrombolysis), healthcare trajectories, the duration of hospital stay, complementary examinations, and vital status at hospital discharge were also reported.

### Statistical analysis

Descriptive results were expressed as percentages for categorical variables, and as means +/− SD or medians [IQR] for continuous variables, as appropriate. Qualitative variables were compared with Chi2 test, and quantitative variables with Student test or non-parametric tests, as appropriate. Statistical analyses were performed with SAS software, version 9.4. A value of 5% was chosen to determine statistical significance.

## Results

### Baseline characteristics

Overall, 301 patients were included in the 3 centers in French Guiana. Three patients with subarachnoid hemorrhage were excluded for the present study. The mean age of the remaining 298 patients was 62.2 years (SD = 14.5). There was a majority of males (63.7%). Over half of the population was born abroad (52%). The main origins were Haiti 41.2%, Suriname 22.5%, Brazil 10.9%, Guyana 5.8%, and St Lucia 5.1%.

**Fig. 1:**
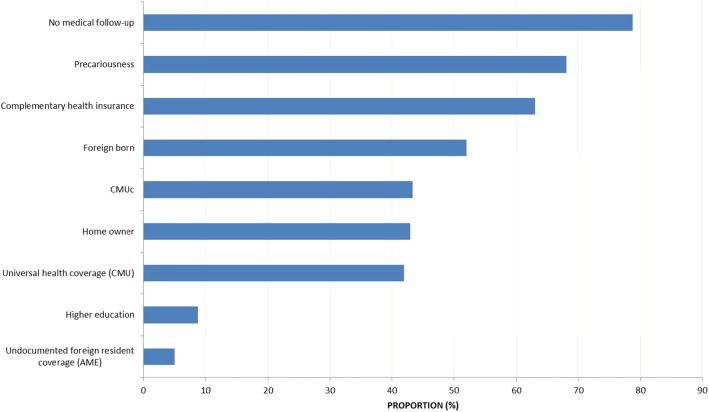
Sociodemographic characteristics of strokes in French Guiana

More than half of the patients (54.2%) lived in a marital relationship, 42.9% were home owners, 8.7% had a high-school diploma, and 68.1% were deprived. Overall, 84.5% had health insurance coverage and among them 41.9% had CMU (universal health coverage for legal residents with annual income under a minimum threshold). Socio demographic characteristics are shown in Fig. 1.

Few patients had consulted a health professional during the past 12 months: there had been no consultation of a general practitioner for nearly a quarter of patients, no dentist consultation for 82.5% of patients and nearly 75% of patients had not consulted any specialist in the past year. Overall, 64.2% of patients in French Guiana lived less than 15 min from a general practitioner, and 45% lived less than 15 min from a hospital.

### Clinical characteristics at baseline and pre-morbid treatment

Most strokes were ischemic strokes (79%). Demographic characteristics and preexisting conditions did not significantly differ between ischemic and hemorrhagic stroke, except for diabetes and prior history of transient ischemic episode, which were more common for ischemic strokes (*P* = 0.003), and alcohol consumption ≥2 drinks/day, which was more common for hemorrhagic strokes (*P* = 0.02).

Table 1 shows that high blood pressure was the most frequent risk factor, followed by diabetes, smoking, obesity and hypercholesterolemia.

**Table 1:** Socio-demographic and baseline characteristics according to stroke mechanism in French Guiana. India study-French Guiana

Characteristics	All patients *N* = 298	Ischemic stroke *N* = 234 [78.5%]	Intracerebral hemorrhage *N* = 64 [21.4%]
*Demographic characteristics*			
Age			
< 50	60 (20.1)	43 (18.3)	17 (26.5)
50–65	106 (35.5)	86 (36.7)	20 (31.2)
65–80	96 (32.2)	77 (32.9)	19 (29.6)
≥80	36 (12)	28 (11.9)	8 (12.5)
Male gender	190 (63.7)	146 (62.3)	44 (68.7)
*Preexisting conditions*			
BMI±			
< 20	16 (5.3)	11 (4.7)	5 (7.8)
20–25	82 (27.5)	60 (25.6)	22 (34.3)
25–30	82 (27.5)	68 (29)	14 (21.8)
> = 30	52 (17.4)	41 (17.5)	11 (17.1)
Unknown	66 (22.1)	54 (23)	12 (18.7)
Hypertension±	208 (70.2)	164 (70.3)	44 (69.8)
Diabetes±	74 (24.9)	67 (28.7)	7 (10.9)
Hypercholesterolemia±	50 (17)	42 (18.1)	8 (12.7)
Chronic heart failure±	16 (5.4)	12 (5.1)	4 (6.2)
Alcohol ≥ 2 drinks/day±	52 (17.9)	35 (15.2)	17 (27.8)
Tobacco status±			
Current smoker	64 (21.6)	53 (22.7)	11 (17.7)
Previous smoker	48 (16.2)	37 (15.8)	11 (17.7)
Non smoker	183 (62)	143 (61.3)	40 (64.5)
Atrial fibrillation±	19 (6.5)	17 (7.4)	2 (3.1)
Prior medical history			
Prior transient ischemic attack	33 (11)	33 (14.1)	0
Prior myocardial infarction	8 (2.6)	5 (2.1)	3 (4.6)
Prior angina pectoris	9 (3)	6 (2.5)	3 (4.6)
Prior history of lower limb arteriopathy	13 (4.3)	13 (5.5)	0
Prior history of vascular surgery	7 (2.3)	7 (2.9)	0
Prior history of angioplasty	7 (2.3)	6 (2.5)	1 (1.5)
Pre-morbid treatment			
Antiplatelet therapy	41 (13.7)	34 (14.5)	7 (10.9)
Anticoagulant therapy±	19 (6.4)	10 (4.3)	9 (14)
Antihypertensive drug±	153 (51.6)	120 (51.7)	33 (51.5)
Statin±	38 (12.7)	31 (13.3)	7 (10.9)
Oral antidiabetic drug±	48 (16.2)	44 (18.9)	4 (6.2)
Psychotropic drug±	15 (5)	9 (3.8)	6 (9.3)
Insulin±	24 (8)	21 (9)	3 (4.6)
Oral contraception ±(woman only)	7 (6.4)	7 (7.9)	0
Substitution hormonotherapy ±(woman only)	2 (1.9)	0	2 (1.9)
Ischaemic stroke classification (*n* = 234)			
Lacunar		57 (24.3)	
Large-artery		38 (16.2)	
Cardioembolism		28 (11.9)	
Other		111 (47.4)	
Admission NIHSS score±			
0–3	89 (31.4)	75 (33.7)	14 (22.9)
4–9	97 (34.2)	84 (37.8)	13 (21.3)
≥10	97 (34.2)	63 (28.3)	34 (55.7)
mRS at discharge±			
0	40 (14.3)	37 (17.1)	3 (4.8)
1–2	102 (36.6)	88 (40.7)	14 (22.5)
3–4	96 (34.5)	67 (31)	29 (46.7)

± missing values: BMI = 66; HTA = 2; Diabetes = 1; Hypercholesterolemia = 4; Chronic heart failure = 3; Alcohol = 8;Tobbaco = 3; AF = 6; anticoagulant = 2; antihypertensive = 2; statin = 1; oral antidiabetic = 2; psychotropic = 1; insulin = 1; oral contraception = 2; Sub hormonotherapy = 4; pre stroke mRS = 1; NIHSS at admission = 15; Barthel score = 2

The mean age of discovery of diabetes was 57.3 years (the information was available for 85% of diabetic patients).

Antihypertensive drugs were the most frequent pre-stroke treatment (51.6%), however 28.4% of patients had no treatment despite knowing they had high blood pressure. Oral antidiabetic drugs were most frequent in patients with ischemic stroke (19% vs 6%, *P* = 0.01), whereas anticoagulant therapy were most frequent in patients with intracerebral hemorrhage before their first stroke (14% vs 4%, *P* = 0.004).

The pre-stroke mRS was 0 for 258/298 (86.8%) patients, 1–2 for 17/298 (5.7%) patients, and 22/298 (7.4%) patients; 281/298 (94.9%) had a pre-Barthel score of 100. The admission NIHSS score and the ischemic stroke classification are also presented in Table 1. The NIH score showed that severe strokes were more frequent in patients with intracerebral hemorrhage (than in patients with an ischemic stroke (55.7% vs 28.3%, *P* = 0.0003).

### Diagnostic work-up and patient care (Table 2)

Care pathways are presented in Table 2.

**Table 2:** Patterns of management according to stroke mechanism. INDIA study-French Guiana

Characteristics	All patients *N* = 298 (%)	Ischemic stroke *N* = 234 [78.5%] (%)	Intra-cerebral hemorrhage *N* = 64 [21.4%] (%)
Thrombolysis*	32 (10.7)	32 (13.6)	0
During in-hospital stay			
Medical treatment			
Antiplatelet therapy ± *	181 (60.9)	181 (77.6)	0
Antihypertensive drug ±	76 (25.8)	61 (26.5)	15 (23.4)
Heparin ±	129 (43.8)	122 (53)	7 (10.9)
Injectable nicardipine ±	98 (33.2)	55 (23.8)	43 (67.1)
Insulin ±	42 (14.1)	38 (16.3)	4 (6.20)
Oral anti diabetics ±	16 (5.4)	16 (6.9)	0
Urinary catheter	35 (11.7)	19 (8.1)	16 (25)
Other examinations			
CT scanner	253 (84.9)	193 (82.4)	60 (93.7)
MRI	179 (6)	152 (64.9)	27 (42.1)
Ultrasonography of the supra-aortic arteries	145 (48.6)	138 (58.9)	7 (10.9)
Angio-scanner	169 (56.7)	139 (59.4)	30 (46.8)
ECG±	294 (98.9)	233 (99.5)	61 (96.8)
Holter±	113 (38.1)	110 (47.2)	3 (4.7)
Trans-oesophageal ultrasonography	17 (5.7)	17 (7.2)	0
Trans-thoracic echocardiography±	225 (75.7)	190 (81.2)	35 (55.5)
MRI as first radiological examination	110 (36.9)	92 (39.3)	18 (28.1)
Had both MRI and scanner	135 (45.3)	112 (47.9)	23 (35.9)
After discharge			
Medical treatment			
Antiplatelet therapy±	197 (72.1)	196 (87.8)	1 (2)
Anticoagulant therapy±	62 (22.7)	51 (22.8)	11 (22)
Antihypertensive drug±	226 (82.7)	182 (81.6)	44 (88)
Statin±	187 (68.5)	180 (80.7)	7 (14)
Non-medical treatment			
Speech therapy±	42 (15.9)	33 (12.5)	9 (18.7)
Physical therapy±	136 (51.5)	106 (49.3)	30 (61.2)
Care pathways– admitted in …			
UNV	1		
Neurology±	181 (61.3)	144 (62)	37 (58.7)
Short stay unit (UHCD)±	177 (60)	135 (45.7)	42 (66.6)
ICU±	11 (3.7)	2 (0.86)	9 (14.2)
Medecine±	65 (22)	53 (22.8)	12 (19)
Otherunits±	43 (14.5)	37 (15.9)	6 (9.5)
Follow up checkup programmed ±	188 (73.4)	152 (73)	36 (75)
Follow up checkup done at hospital	139 (50.3)	109 (48.6)	30 (57.6)
Follow up checkup done: Examinations done after discharge			
MIR	33 (23.70)	19 (17.40)	14 (46.60)
Arteriography	3 (2.10)	2 (1.80)	1 (0.72)
Trans-esophagealultrasonography	10 (7.10)	10 (9.10)	0
Transthoracicultrasonography	4 (2.80)	4 (3.60)	0
Holter±	12 (8.70)	11 (10.10)	1 (3.30)

± Missing values:

∙ During care: Antiplatelet therapy = 1; Antihypertensive drug = 4; Heparin = 4; Injectable nicardipine = 3; Insulin = 1; Oral anti diabetics = 2; ECG = 1; Holter = 2; Trans-thoracic echocardiography = 1

∙ At discharge: Antiplatelet therapy = 25; Anticoagulant therapy = 25; Antihypertensive drug = 25; Statin = 25; Speech therapy = 34; Physical therapy = 34

∙ Care pathway: Neurology = 3; Short Stay Unit = 3; ICU = 3; Medicine = 3; other = 3; Follow up check-up programmed = 42; Follow up check-up done = 22

∙ Follow up checkupdone: Holter = 1

*significant *p* value

Medical treatments administered during hospital stay differed between ischemic stroke and intracerebral hemorrhage (Table 2). Among patients with ischemic stroke, only 13.6% of patients received thrombolysis. The most administered treatment in patients with ischemic stroke during hospital stay were antiplatelet therapy, heparin, antihypertensives and injectable nicardipine (Table 2). Injectable nicardipine was the most frequently prescribed treatment in patients with intracerebral hemorrhage.

Cerebral CT scan was performed in 85% of patients (94% of patients with intracerebral hemorrhage) and MRI in 60% of patients. Ultrasonography of the supra-aortic arteries and angioscanner were performed in 59% of patients with ischemic stroke, and trans-thoracic echocardiography in 80% of them. Holter EKG was performed in nearly half of ischemic stroke.

After discharge among patients with ischemic stroke, 88% received antiplatelet therapy, 82% antihypertensives, and 81% statins. For patients with intracerebral hemorrhage, antihypertensives were the most prescribed therapy after discharge (88%), followed by anticoagulants (22%). Among patients for whom the information was available (*n* = 213), the median delay between the first symptoms and hospital admission was higher for ischemic stroke (3.4 h [IQR: 1.3–11.2] than for intracerebral hemorrhage (1.9 h [IQR: 1.2–3.6]), *p* = 0.02. The median duration between symptoms onset and thrombolysis was 3.3 h [IQR: 2.5–4] and the median duration between admission and thrombolysis was 1.9 h [IQR:1.4–2.8]. Among patients admitted in less than 4.5 h after an ischemic stroke, only 34.8% (31/89) received thrombolysis. Overall 89 (38%) patients with ischemic stroke were admitted less than 4.5 h after the first stroke symptoms.

### Outcomes at hospital discharge by stroke mechanism

The median length of stay was 11 days [IQ: 7–17]. In-hospital mortality was greater in patients with intracerebral hemorrhage than patients with ischemic stroke (18.7% vs 4.2%, *P* = 0.0001). The mRsat discharge by type of stroke is presented in Table 1, it was significantly more severe in hemorrhagic than in ischemic strokes (*P* = 0.0002).

Upon discharge 156 patients (52.3%) went back home, 98 patients (32.9%) were transferred in a rehabilitation ward and 22 patients (7.4%) were transferred in another hospital for further care.

## Discussion

Although it was known that the incidence of stroke was high, the present study provides the first epidemiological data on strokes in French Guiana. The comparison of the results of the present descriptive study with those of French stroke registries showed that the proportion of ischemic to hemorrhagic strokes and the frequency of risk factors were similar in French Guiana and France, but underlined that strokes in French Guiana affected younger patients, and more males than in mainland France. The mean age of 62.2 years (±14.5) was also lower than what is usually described in South America (65.8(±12.5)), and was on par with what has been described in South Asia, South East Asia, China and Africa [18]. The proportion of strokes before 45 years of age was 12.6%. This was significantly higher than in France or South America but lower than in South Asia, South East Asia or Africa [18]. The younger age of patients in French Guiana may be linked to difficulties in access to care and delays in diagnosis and treatment of hypertension, diabetes, and/or hypercholesterolemia. However, there may be other factors in French Guiana that underlie this observation: genetic factors, a different ethnic mix in French Guiana than in mainland France, or early life nutritional deficiencies [19] or the high prevalence of lead poisoning, which are risk factors for hypertension and perhaps strokes [20, 21]. Over half of patients were foreign, which seems high but corresponds to the demographic situation among adults in the general population of French Guiana [22]. The socio-economic data of French Guiana in this study reflect the current situation of this territory, which faces greater levels of social inequality than in mainland France [23, 24].

The study of risk factors for stroke showed that hypertension was the main risk factor as in mainland France [6, 25, 26], and that past and current smoking was the second most frequent risk factor with a similar proportion than what is observed in France (58%) [27]. followed by diabetes for 25% of patients.

In the present sample, there was no significant difference in the proportion of hemorrhagic and ischemic strokes relative to what is described in mainland France [28]. The modified Rankin score upon discharge was significantly higher in French Guiana showing patients with more severe stroke consequences at 1 month than in Western Europe and North America (At 1 month: French Guiana vs Europe-North America: 9.16% vs 1.6%, respectively for death and 20.51% vs 7.1%, respectively for moderately and severe disability, (mRS 4–5)) [18]. This is preoccupying given the structural deficit in terms of available downstream structures for the rehabilitation of patients. However there were more patients in French Guiana with a mRS score < 2 after discharge than in Brazil (French Guiana vs Brazil: 50.9% vs 27.7%, respectively) [29].

In terms of emergency care, patients in French Guiana generally had similar delays between symptoms and admission than patients in mainland France (2 h58[1.55–8]) [30], which was somewhat surprising because the territory of French Guiana is very large and some parts are very isolated from the emergency facilities. It is not possible to rule out a bias where these patients would tend to arrive later, with more severe presentations, without a next of kin, and thus may not have been included in the study. After admission there was a longer delay between admission and thrombolysis in French Guiana 1.9 h [1.4–2.8] relative to mainland France (1.1[0.8–1.6]) [30]. However, it is noteworthy that the proportion of missing data on the above time intervals was not negligible. Despite this difference, the overall proportion of patients receiving thrombolysis was not much different from that observed in France (14.3%) [30],21.9% in the Netherlands, 16.3% in theUSA [31, 32], but clearly higher than countries in South America like Brazil (1.1% in Fortaleza, 2.7% in São Paulo) [29, 33]. This proportion, which increases rapidly with the progressive structuration of emergency care, should be continuously monitored because in French Guiana, repeated staff shortages are likely to have adverse consequences of on this performance indicator.

Regarding diagnostic procedures, all patients had initial imagery, as in most countries, 38.1% had a holter monitoring which is higher than western average [18], nearly 75.7% had transthoracic echocardiography, a proportion that is much higher than international averages (11.5%) [18, 29]. The importance of baseline examinations for stroke has been demonstrated for the identification of dysfunction and precise diagnosis [34, 35]*.* We can also note that there was a correct completion rate for the EKG, ETT and ultrasonography of the supra-aortic arteries, which shows that patients were properly investigated.

One of the study strengths is that it included patients prospectively. Nevertheless, the study has a number of limitations: There were missing values in some of the studied variables which led to exclude these variables from the analysis; The sample size was small, and this may have reduced the precision of estimates and the power for the repeated comparisons and an inflated alpha risk; another potentially important limitation was the possibility of a recruitment bias for patients arriving too late and having no next of kin present, such patients could not be included in the study and thus lead to more optimistic estimates of thrombolysis rate and stroke-admission intervals. Finally, the comparisons of aggregated data are instructive but further studies should conduct multivariate analyses of individual data to control for interactions and confounding.

## Conclusion

Overall these results show that beyond the quantitative singularity of the high incidence of stroke in French Guiana shown in other studies [28], the patients also have an atypical epidemiologic profile, greater mortality possibly due to delays in patient care relative to Europe and North America and more severe consequences 1 month after the stroke. The potential implications of these results cover different domains of health: primary prevention through health promotion of balanced nutrition and exercise, health education should inform about the need and procedure to get screened for hypertension and diabetes and the importance of proper follow-up. Although there have already been efforts in French Guiana to inform about the signs of stroke and what one should do, the 15 emergency medical service free number is not sufficiently known [36] and the public should be educated to call this number without delay to avoid loss of time. Outreach efforts should aim to increase screening for stroke risk factors. Hence, in the 1970s proactive population-based interventions in the USA, screening for hypertension and diabetes have shown a rapid 26% reduction of cardiovascular mortality [37, 38]. The absence of neurovascular unit should be corrected in order to offer the same chances to ischemic patients as those in mainland France. The lack of downstream structures to care for patients that are generally more severe also seems a major priority. Finally, although diagnostic delays and insufficient access to care are highly plausible determinants of these differences with mainland France, further epidemiological research should look for other determinants (genetic, nutritional, and/or toxic) that may further increase the stroke risk in French Guiana.

## Data Availability

Researchers wishing to access the data should first obtain permission from the Commission Nationale Informatique et Libertés. For this, the research proposal must be submitted to the CNIL following the indication on their website (CNIL https://www.cnil.fr/en/home). Once the approval of the CNIL has been obtained by the researchers, the anonymized and encrypted database will be sent by the research organization of CIC-EC (cicec@ch-cayenne.fr). If you have any questions, it is possible to contact Marilyne ABIVEN (director of the indirect right of access service department of the CNIL) at mabiven@cnil.fr.

## Abbreviations

CMU: Couverture Maladie Universelle
INDIA: INégalités sociales et pronostic des accidents vasculaires cérébraux à DIjon et en Antilles-Guyane
IQR: Interquartile Range
mRS: Modified Rankin Scale
NIHSS: National Institute of Health Stroke Scale.
PCP: Primary Care Physician
SAH: Subarachnoid hemorrhage
SD: Standard Deviation
USA: United States of America
